# Bone strain index as a predictor of further vertebral fracture in osteoporotic women: An artificial intelligence-based analysis

**DOI:** 10.1371/journal.pone.0245967

**Published:** 2021-02-08

**Authors:** Fabio Massimo Ulivieri, Luca Rinaudo, Luca Petruccio Piodi, Carmelo Messina, Luca Maria Sconfienza, Francesco Sardanelli, Giuseppe Guglielmi, Enzo Grossi

**Affiliations:** 1 UO Medicina Nucleare, Fondazione IRCCS Ca’ Granda Ospedale Maggiore Policlinico, Milano, Italy; 2 TECHNOLOGIC Srl, Lungo Dora Voghera, Torino, Italy; 3 Milano, Italy; 4 UO Radiologia Diagnostica e Interventistica, IRCCS Istituto Ortopedico Galeazzi, Milano, Italy; 5 Diagnostica per Immagini e Radioterapia, Dipartimento di Scienze Biomediche per la Salute, Università degli Studi di Milano, Milano, Italy; 6 Radiologia e Diagnostica per Immagini, IRCCS Policlinico San Donato, Piazza Edmondo Malan, San Donato Milanese (MI), Italy; 7 Dipartimento di Medicina Clinica e Sperimentale, Università degli Studi di Foggia, Viale Luigi Pinto, Foggia, Italy; 8 Villa Santa Maria Foundation, Tavernerio (CO), Italy; Humanitas Clinical and Research Center - IRRCS, ITALY

## Abstract

**Background:**

Osteoporosis is an asymptomatic disease of high prevalence and incidence, leading to bone fractures burdened by high mortality and disability, mainly when several subsequent fractures occur. A fragility fracture predictive model, Artificial Intelligence-based, to identify dual X-ray absorptiometry (DXA) variables able to characterise those patients who are prone to further fractures called Bone Strain Index, was evaluated in this study.

**Methods:**

In a prospective, longitudinal, multicentric study 172 female outpatients with at least one vertebral fracture at the first observation were enrolled. They performed a spine X-ray to calculate spine deformity index (SDI) and a lumbar and femoral DXA scan to assess bone mineral density (BMD) and bone strain index (BSI) at baseline and after a follow-up period of 3 years in average. At the end of the follow-up, 93 women developed a further vertebral fracture. The further vertebral fracture was considered as one unit increase of SDI. We assessed the predictive capacity of supervised Artificial Neural Networks (ANNs) to distinguish women who developed a further fracture from those without it, and to detect those variables providing the maximal amount of relevant information to discriminate the two groups. ANNs choose appropriate input data automatically (TWIST-system, Training With Input Selection and Testing). Moreover, we built a semantic connectivity map usingthe Auto Contractive Map to provide further insights about the convoluted connections between the osteoporotic variables under consideration and the two scenarios (further fracture vs no further fracture).

**Results:**

TWIST system selected 5 out of 13 available variables: age, menopause age, BMI, FTot BMC, FTot BSI. With training testing procedure, ANNs reached predictive accuracy of 79.36%, with a sensitivity of 75% and a specificity of 83.72%.

The semantic connectivity map highlighted the role of BSI in predicting the risk of a further fracture.

**Conclusions:**

Artificial Intelligence is a useful method to analyse a complex system like that regarding osteoporosis, able to identify patients prone to a further fragility fracture. BSI appears to be a useful DXA index in identifying those patients who are at risk of further vertebral fractures.

## Introduction

Osteoporosis is a metabolic bone disease characterised by a reduction in bone mass and deterioration in texture and architecture of bone, leading to fragility fractures [1]. Osteoporotic fragility fractures cause significant morbidity and mortality in the elderly population. Whereas hip fractures almost inevitably lead to hospitalisation, vertebral fractures may even be asymptomatic, but patients present a clinical disability that is correlated with the number and the severity of the fractures [2]. The diagnosis of osteoporosis is based on the measurement of the Bone Mineral Density (BMD) by Dual X-ray Absorptiometry (DXA) [3]. Areal BMD (the bone mineral content measured by DXA and divided by the bone area in square centimeters) is responsible for about two-thirds of bone strength, and fracture risk increases proportionally with the reduction of BMD [4]. However, assessment of BMD does not completely detect fractured patients, because there is an overlap of BMD in patients with or without fractures, being the fractured ones more numerous in the normal/osteopenic than in the osteoporotic group [5]. This finding raises the question of the clinical assessment of fracture risk with BMD alone due to its lack of sensitivity [6]. So there is a need for other tools that can predict fracture risk in addition to BMD, for the evaluation of bone micro-architectural and textural structure [7]. Its direct examination can be done invasively by bone biopsy and histomorphometry or indirectly by some imaging techniques as high-resolution peripheral quantitative computed tomography or magnetic resonance imaging [8]. These procedures are, however, expensive or with high ionising radiation dose to the patient, and therefore not accessible for screening. For these reasons other DXA indexes have been developed for bone texture analysis. The Trabecular Bone Score (TBS) is a tool correlated with histomorphometric bone parameters and can be performed during a lumbar spine DXA scan [9] to evaluate local variations in grey levels from the image. Previous studies showed that TBS could predict the fracture risk partially independently from BMD [10].

A new DXA-based parameter has been recently created, with the name of Bone Strain Index (BSI) [11]. This tool represent the analysis of a deformation index that is obtained by the use of finite element analysis from DXA images, and it is consistent with the mathematical model called Finite Element Model (FEM) [12]. Finite Element Models are based on the idea that a complex object can be divided in smaller and simpler elements to simplify the problem-solving. In bone structural analysis FEM can be used to solve mechanical problems and identify the bone area most prone to higher stresses, the bone strains and fracture risk. Many models have been proposed to describe the mechanical behaviour of bones [13, 14] depending mainly on base image type, anatomical site, material properties assigned to the bone and loading conditions.

BSI calculation is obtained using a triangular mesh designed on the bone segmented by the DXA software. Mechanical properties are assigned at each element depending on local BMD and anatomical site, according to Morgan et al. formulas [15]. In case of the lumbar spine the loading force applied to each vertebra is calculated following simulation data provided by Han study in standing conditions [16] and uniformly distributed on the upper facet of each vertebra, whereas the lower side is used as a constraint. In the case of hip scans, the loading and the constraints were characterized by head and distal femur forces applied on the greater trochanteric area [17].

In both cases, BSI represents the average equivalent strain in the regions of interest identified by DXA software (e.g. vertebra regions for lumbar scans; neck, trochanteric and intertrochanteric regions for hip scans). BSI can provide a quantitative description of the strain distribution inside the considered bone segment that is not offered by the other cited DXA indexes, BMD and TBS. Being BSI an index of the strain concentration, higher values indicate a significant risk condition, whereas lower values indicate a protected status. In recent clinical studies, BSI appeared to be useful to identify osteoporotic patients with higher fracture risk [18], to characterise patients affected by secondary osteoporosis [19, 20] and to predict fracture [16].

Osteoporosis is a multi-factorial pathology, in which the physician has to take into account several factors that are linked between each other in a complex network; this setting may result not easy to investigate using the classical statistical analysis. In order to face such complex scenario, a new mathematical methodology based on an Artificial Neural Network Analysis (ANNs) named Auto Contractive Map has been applied to analyse a database of osteoporotic patients [18, 15]. ANNs represents adaptive computational procedures inspired by human brain working processes, specifically arranged to figure out non-linear questions and unveil faint associations among variables [21]. Using a learning process based on adaptive ways (obtaining from existing data the information needed to complete a particular aim and generalizying the knowledge acquired), the ANNs represent a strong tool for data analysis even when samples are relatively small. The use of Auto-CM approach has growth in recent years and has been applied to several diseases, showing its potential in the identification of strong connection among clinical, laboratory and new "omics" biomarkers [18, 22–24].

In this paper, we have used a combination of two ANNs approaches, one (TWIST system, see S1 File) aiming to develop a predictive model to be applied at the individual level to predict the future development of vertebral fractures and the second (the Auto Contractive Map—Auto-CM) [25, 26] to provide additional knowledge regarding the intricate biological connections among the densitometric variables we examined and the two conditions (further fracture vs not fracture), with a particular focus on BSI.

## Materials and methods

### Patients

This work is a prospective longitudinal multicentric study conducted at Fondazione IRCCS Ca’ Granda Ospedale Maggiore Policlinico of Milan, IRCCS Istituto Ortopedico Galeazzi in Milan, and IRCCS Policlinico San Donato, San Donato Milanese, Italy. Among the outpatients ongoing to the cited hospitals for osteoporosis, 172 consecutive women found to have a vertebral fracture were enrolled. Inclusion criteria were having a spine X-ray and a femur and spine DXA scan. Exclusion criteria were the presence of bone diseases or pharmacological treatments that may interfere with bone metabolism, as well as previous traumatic and pathological fractures. Treatment for osteoporosis was not an exclusion criterion.

All patients had lumbar and femoral DXA scans every two years to obtain data about femoral and lumbar BMC, BMD and BSI, plus a spine X-ray for spine deformity index (SDI). The presence of vertebral fractures was evaluated by means lateral x-rays using the Genant’s semi-quantitative approach. A radiologist with 10 years of experience in the imaging of osteoporosis assessed the presence of fractures. SDI was calculated by summing the semi-quantitative score (from 0 to 3) of each vertebra from T4 to L4 (minimum score = 0, maximum score = 39) [27]. The presence of an incident re-fracture was defined in case of a one point increase of SDI value observed at follow-up. Patients performed both DXA scan and X-ray spine exam at each clinical check.

Anagraphic, anthropometric and clinical data were collected. Written informed consent was signed by all patients for the management of their sensitive data for scientific research each time they entered the hospital. Local Ethical Committees approval was obtained (Comitato Etico Milano Area 2. Protocol N 2.0 BQ. 265_2017, 13th June 2017 for IRCCS Fondazione Ca’ Grande Ospedale Maggiore Policlinico, Milan, Italy. Comitato Etico San Raffaele. Studio clinico 2.0 BQ, version 4.0, 8th August 2019, for IRCCS Istituto Ortopedico Galeazzi, Milan and IRCCS Policlinico San Donato, San Donato Milanese, Italy).

### Methods

#### DXA data acquisition

Bone density was assessed with DXA, using a Hologic Discovery A for Fondazione IRCCS Ca’ Grande Ospedale Maggiore Policlinico and IRCCS Policlinico San Donato, and a Hologic QDR-Discovery W for IRCCS Istituto Ortopedico Galeazzi.

DXA-dedicated technicians with experience in the technique performed all exams in accordance to the International Society for Clinical Densitometry (ISCD) official positions [28]. All patients were scanned by DXA both at lumbar spine and at proximal femur. Fractured vertebrae were not considered in the analysis. BMD was obtained automatically as the ratio between BMC (g) and the scanned area (cm2); BSI was automatically derived with a dedicated external software from the same area of spine scans.

**Fig 1:**
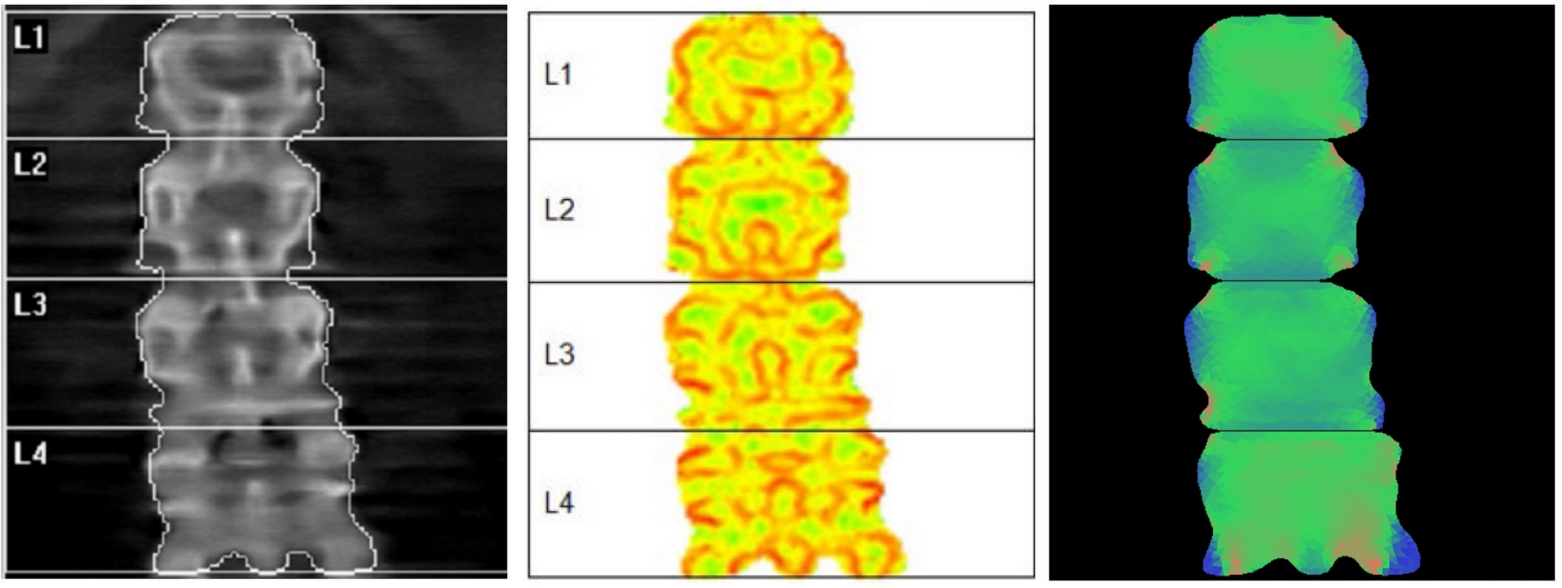
Example of a DXA image showing the three parameters that we used: BMD, TBS and BSI analysis.

BSI software generates a Finite Element Analysis on DXA images sent through DICOM protocol and provides the Bone Strain Index in less than 10 seconds. The triangular mesh is created relying on bone segmentation performed by the operator on the DXA software. The calculation of material properties is based on experimental formulas provided by Morgan et al. 2003 [29], whereas the definition of boundary conditions depends on the anatomic site. For the lumbar region, standing condition are simulated [30] and force is automatically applied to the upper surface of each vertebra, while the lower surface is constrained. For the femoral region, the force is calculated in falling condition [17] and applied to the greater trochanter, whereas constraints are placed on the head and the lower femoral shaft. TBS is automatically calculated form the lumbar scan (Medimaps, Geneva, Switzerland). Fig 1 swows an example of the three images obtained at lumbar spine; from left to right a DXA scan (BMD total value = 0.83), a TBS scan (TBS L1-L4 = 1.240) and a BSI scan (Lumbar BSI = 2.01).

#### X-ray data acquisition

Patients underwent an anteroposterior and lateral X-ray of the spine to investigate the presence of vertebral fractures (VFs) at the first baseline examination and at the end of the follow-up. The SDI was calculated using the semi-quantitative approach [27, 31] in order to obtain a correct definition of osteoporotic vertebral fracture [32].

#### Predictive analysis with supervised Artificial Neural Networks (ANNs)

In this study we applied novel intelligent system, by combining artificial neural to evolutionary algorithms. Supervised ANNs [33] was used to create a model with the ability to predict the diagnostic class (further fracture vs not) starting from the densitometric data alone, with high degree of accuracy. Supervised ANNs are networks that are capable to learn about a condition using examples, with a training phase in which they calculating an error function and therefore correcting the connection strengths to minimise the error. The learning method of the supervised ANNs is able to make their output coincide with a pre-established target. In general, the mathematic formula of ANNs is y = f(x,w*), in which w* represents the set of parameters that approximate the function at its best.

Pre-processing of our data was done with the use of a re-sampling system called “TWIST”, created by the Semeion Research Centre (see S1 File). The TWIST system combines two systems: the T&T and the IS [34]. The T&T system relies on a data re-sampling technique capable of arranging the reference sample into various sub-samples that hold a similar density function probability. With this approach, data under analysis are separated into two or more sub-samples, with the purpose of more effective train, test and validation of the ANN models. The IS is an evolutionary system capable of reducing the quantity of data while preserving the most important information contained within the dataset. The combination of these two systems helps in solving two frequent problems when using Artificial Neural Networks: from one side the best splitting of training and testing subsets, which contains a balanced distribution of outliers; from the other side, the best selection of variables containing the highest amount of information that are relevant to the investigated problem. Both T&T and IS are based on a Genetic Algorithm called the Genetic Doping Algorithm (GenD), which was created at Semeion Research Centre [35]. The TWIST system has already been applied to different medical scenarios [36–40], and we describe it more in detail in the S1 File. After this, the most relevant features are selected and, simoultaneously, the training-testing set are developed with a function of probability distribution comparable to those who provided the best classification results. A supervised Multi-Layer Perceptron, based on four hidden units, was subsequently used for performing the classification.

#### Data set preprocessing for semantic connectivity map

We transformed the thirteen input variables in 26 input variables constructing for each of them, scaled from 0 to 1, its complement. Consider, for example, the variable LBMD. Absolute natural values range from 0.521 to 1.3. In transformation 1.3, the highest value, becomes 1 and 0.521 becomes 0. All the remaining values are scaled in such new range, so, for example, the value 0.64 becomes 0.15; the value 0.93 becomes 0.53, and so on. The projection of the variable LBMD in the map needs an adequate transformation in order to avoid fuzzy position of variables xin the map. In the complement transformation, by subtracting the scaled value from 1 (e.g. 0 becomes 1), we permit the system to point out and project the fuzzy position of the variable also according to its lower values. This is important because, in non-linear systems, there is a non necessarily symmetric position of high and low values related to a given variable. Therefore, the projection of the original variables usually showed high values, whereas the complement transformation showed low values. In the map, we called these two forms as "high" and "low". This pre-processing scaling is needed to proportionally compare all the variables and to identify the links between each variable when their values tend to be high or low.

In our study we decided to rely on the minimum spanning tree (MST) algorithm as a base to perform variables clustering, creating a semantic connectivity map with the aim to discover hidden relationships not necessarily detectable with other clustering approaches. A detailed description of the working process of MST algorithm is provided in the S1 File. The MST was applied to a similarity matrix obtained with prior probability equation. Prior probability (PP) algorithm takes into account the ratio of probabilistic concordance/discordance between a couple of variables (see S1 File for a detailed description of PP algorithm). Auto-CM algorithm is a fourth-generation ANN, designed at the Semeion Research Center [21, 26]. Finally, at the last part of the training phase the weights of the connections among variables are taken as similarity measures to form the distance matrix.

## Results

Table 1 shows the characteristics of the population studied. Follow up ranged 1–11 years (mean 3.28, SD 2.14, median 4.95).

**Table 1:** Characteristic of the studied population.

Characteristic	Mean	SD	Min	Max
Menopause age	48.49	4.92	33.00	58.00
SDI	3.78	3.43	1.00	20.00
Age	69.35	8.53	44.00	87.00
BMI	24.41	4.42	14.67	42.46
LBMC	35.70	9.69	12.55	60.50
LBMD	0.74	0.12	0.40	1.20
LBSI	2.34	0.63	1.11	4.59
Neck BMC	2.90	0.41	1.72	3.90
Neck BMD	0.58	0.08	0.32	0.81
Neck BSI	1.98	0.46	1.13	3.42
FTot BMC	23.69	4.06	13.48	35.94
FTot BMD	0.69	0.10	0.40	0.98
Ftot BSI	1.73	0.35	1.04	2.96

Table 2 shows the characteristics of the two groups of patients developing (93 pts) and not developing (79 pts) a further vertebral fracture (VF) during the follow-up period. The mean values of the following variables resulted significantly different among women developing a VF in comparison to women without VFs during the follow up: LBMD, LBSI, Neck_BMC, Neck_BMD, Neck_BSI, FTot_BMC, FTot_BMD, Ftot_BSI.

**Table 2:** Characteristics of the two groups of patients, fractured vs not fractured.

	Not re-fractured		Re-fractured		
Characteristic	Mean	SD	Mean	SD	P value
					
Menopause age	48.63	3.90	48.30	5.61	N.S.
SDI	3.89	3.73	3.58	3.04	N.S.
Age	68.38	7.51	70.35	8.95	N.S.
BMI	23.66	4.43	24.91	4.35	N.S.
Lumbar BMC	35.04	9.86	36.11	9.39	N.S.
Lumbar BMD	0.75	0.11	0.74	0.11	N.S.
Lumbar BSI	2.23	0.56	2.43	0.67	N.S.
Neck BMC	2.88	0.39	2.93	0.43	N.S.
Neck BMD	0.58	0.08	0.58	0.08	N.S.
Neck BSI	2.01	0.42	1.95	0.49	N.S.
Total Femur BMC	23.14	3.43	24.14	4.51	N.S.
Total Femur BMD	0.68	0.09	0.7	0.11	N.S.
Total Femur BSI	1.75	0.31	1.71	0.37	N.S.

**Fig 2:**
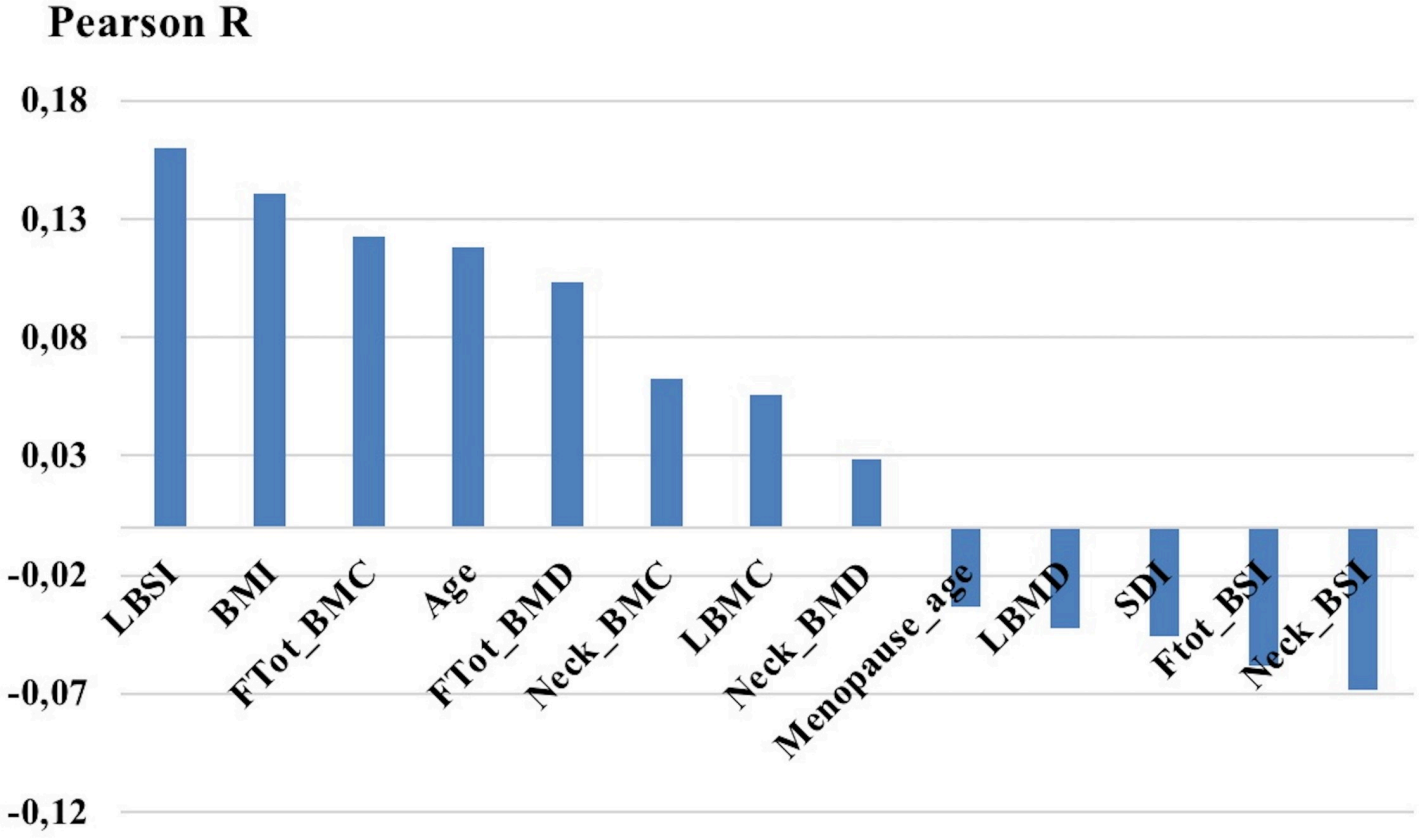
Linear correlation index between patients characteristics and the presence of a further fracture.

Fig 2 shows the linear correlation values between the studied variables and the presence of a VF at follow up. As expected, BSI predisposes to VF at variance with bone mineral contents parameters. In any case, the absolute value of Pearson R is rather low, and this offers a further rationale for the application of ANNs.

The TWIST system selected the following variables which took part in the modelling by artificial neural networks: Menopause age, age, BMI, FTot BMC, Ftot BSI.

Interestingly, the system also selected variables with low linear correlation index like menopause age (-0.033), which would have been almost certainly discarded by linear modelling approaches. A global dataset of 5 inputs and two target attributes was thus generated. After that, two optimal subsets were created to apply the training and testing procedure described in the materials and methods section. Table 3 shows the results obtained by the application of a backpropagation artificial neural networks with eight hidden nodes to the variables selected by TWIST system.

**Table 3:** Predictive results using artificial intelligence systems. The results refer to two testing experiments with training-testing A-B and B-A sequence.

ANN	Records	Fracture YES	Fracture NO	Sensitivity (%)	Specificity (%)	Overall Accuracy (%)	AUC
Feed forward back propagation AB	99	56	43	67.86	83.72	75.79	0.782
Feed forward back propagation BA	99	56	43	82.14	83.72	82.93	0.896
Sum/mean	198	112	86	75	83.72	79.36	0.83

**Fig 3:**
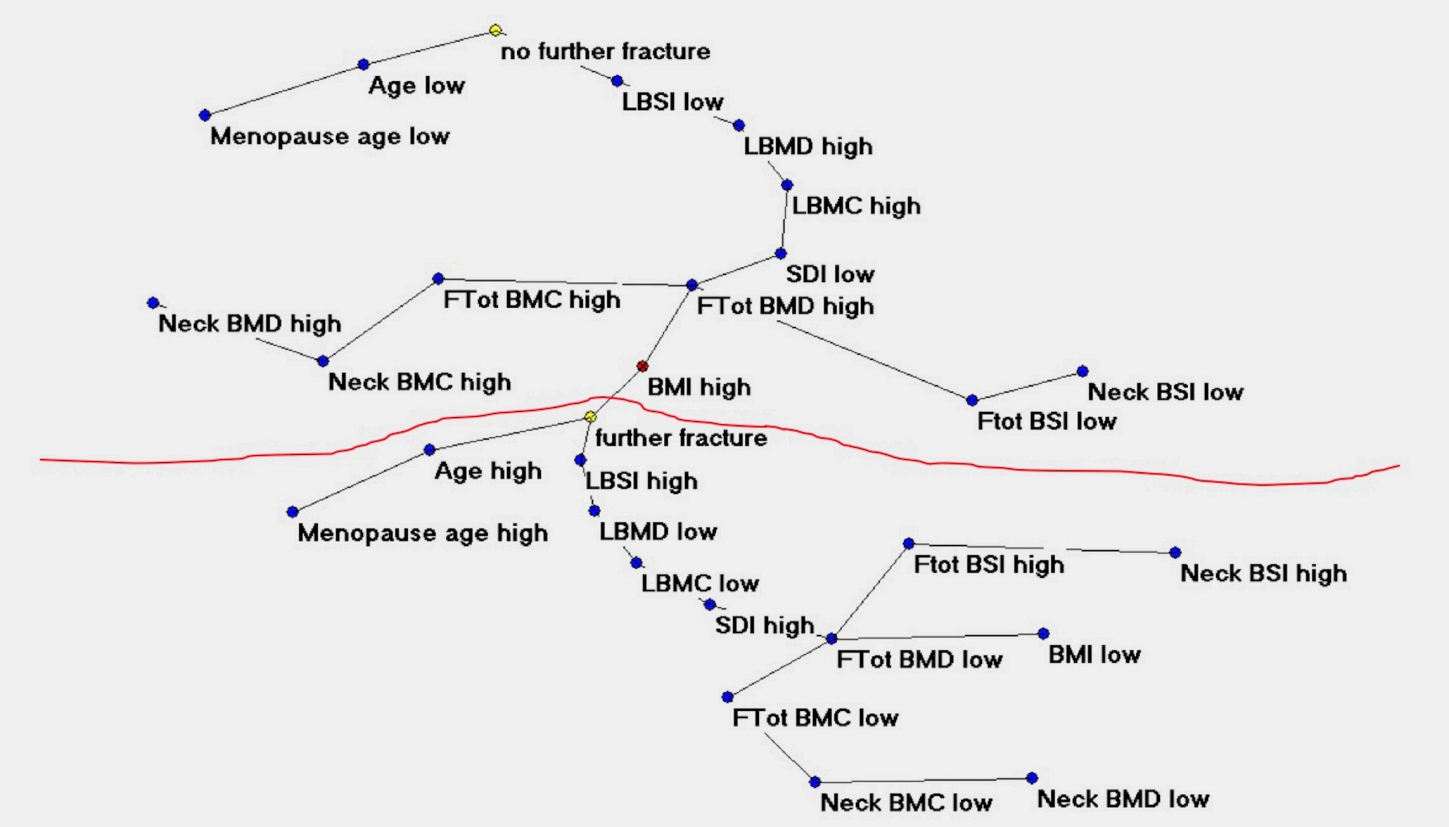
Semantic connectivity map of the studied variables.

The performance of artificial neural network resulted remarkable, with an overall predictive accuracy shown in Table 3 and Fig 3 near to 80%.

The main data set has been divided into two parts (Table 3): a subset A consisting of 90 records (49 with further fractures and 41 without further fractures) and a subset B consisting of 82 records (44 with further fractures and 38 without further fractures). This optimal subdivision has been carried out using an evolutionary algorithm named T&T that builds two sets, Set A and Set B, trying to approximate the same probability density function. Traditional techniques, such as cross-validation, leave-one-out variant and bootstrapping, do not guarantee good results when the global dataset is limited or complex, whose data is hyper-point of an unknown non-linear function: the sub-samples extracted are not always representative of the phenomenon. They do not show the same density function probability. The division into training and testing sets on a random basis not only does not consider the problem of outliers but has consequences in terms of variability of results.

Training and testing validation protocol have been used to compare the classification tasks. It consists of the execution of two independent procedure. The first one, named a-b, uses as training set the previously built subset A and the subset B as a testing set. The second, named b-a, reverses roles using subset B as a training set and subset A as testing. The pair of predictions is then averaged to get a final value. Evaluating the results considering both classifications a-b and b-a prevents the possibility of selecting a particularly favourable sample.

Fig 3 shows the semantic connectivity maps developed by Auto-CM system, illustrating the connections among variables in the not fracture area (no further fracture) and the fracture area (further fracture). Observing the map in Fig 2 with only minimum spanning tree filter we can appreciate which variables act as predisposing factors for further fractures and specifically: high values of BMI, advanced age and high values of LBSI. All three variables are directly connected with the node representing further fracture. On the other hand, protective variables, i.e. variables directly connected with no further fracture are low age, and low values of LBSI.

Fig 3 shows some red links superimposed to the minimum spanning tree indicating areas of the map in which internal loops indicate a high degree of complexity and interconnections among various factors. All variables included in these two “diamonds” interact dynamically.

**Fig 4:**
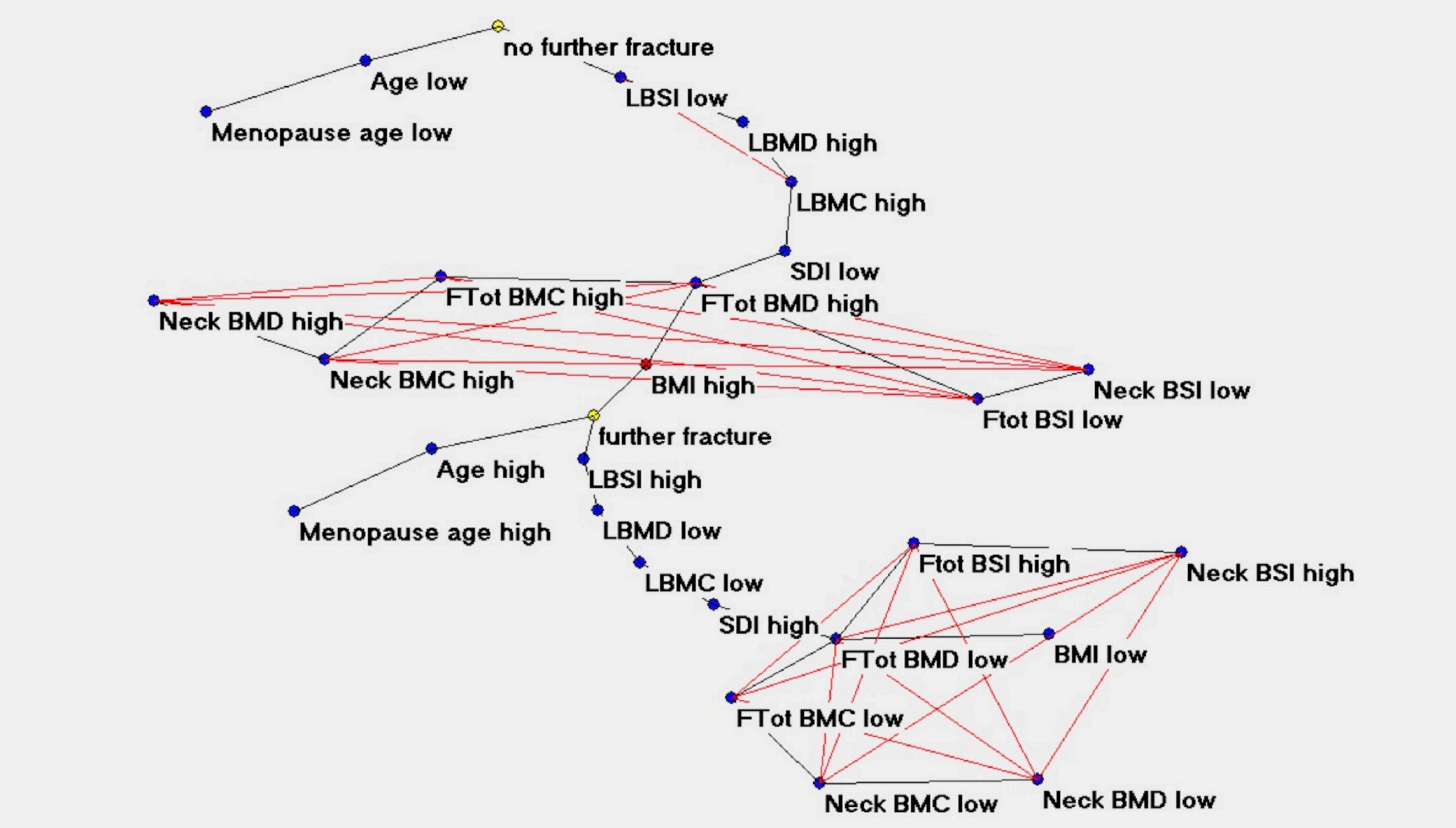
Semantic connectivity map with maximally regular graph.

Fig 4 shows the same connectivity map with the superimposition of maximally regular graph depicting a sort of diamond in which there are multiple interconnections among variables meaning the inherent complexity of the data structure.

## Discussion

Osteoporosis is characterized by an inherent complexity with many factors of different clinical significance interacting towards the possible development of the fragility fracture. One of the significant challenges in the management of the patient affected by possible osteoporotic fracture is identifying, among the many variables related to the fracture risk, those of highest weight in determining the path to the fracture or further fracture event, because the vast number of variables considered can complicate the comprehension of the clinical meaning of the correlations found. For this reason, we used an innovative approach to statistical analysis of our database, called Artificial Neural Network analysis (ANNs) with a robust data mining system, Auto-CM.

In our population of fragility fractured women, we have applied supervised neural network modelling on the baseline variables selected by TWIST system. The analysis highlights a high performance of artificial neural networks that resulted remarkable, with an overall predictive accuracy near to 80%.

In the medical field, the machine learning data mining represents a relatively new method emerging with the advent of genomic and functional data. The available techniques offered by classical statistics like principal component analysis or hierarchical clustering suffer from several drawbacks due to the complexity of possible interactions between risk factors, their non-linear influence on the disease occurrence and the essential stochastic components. Auto-Cm network, a fourth-generation ANNs, arises just to overcome these limitations. Auto-CM has been applied in different medical contexts with impressive results demonstrating the ANNs’ usefulness in easily "untangle the ball of yarn" of complex systems characterised by many variables with different significance [41].

We observed a complex relationship between bone quantity and quality DXA variables with high adaptive weight among the connections (Figs 2 and 3) with the definition of two well distinct clusters: one characterised by a degraded bone quantity (low LBMD) and quality (high LBSI) and the presence of further vertebral fragility fracture (below the red line in the Fig 2), and one characterised by a good bone quantity and quality and the absence of further fracture. It is noteworthy that the first bone variable directly related to the event (further fracture, no further fracture) is LBSI, indicating that degrading in bone strength (LBSI high) is a significant risk factor for further VF, as ultimate step in the pathway leading to the event. This confirms what reported in literature about the role of BSI in characterizing the pathway leading to fragility fracture in post-menopausal women [18]. Conversely, a good bone strength (LBSI low) is a bone variable directly related to good bone status. The variable Age plays a similar role in both the events too, confirming the fact that an increasing age is a significant risk factor for fractures.

It is interesting to note that semantic connectivity map highlights two regions with a dense network of connections, one in the area of no further fracture and another in the area of further fracture. The first highly connected area links variables of high femoral bone quantity and quality DXA indexes, while the other area links the femoral DXA indexes of degraded bone status. It could be argued that for a healthy bone status, where further vertebral fractures unlikely occur, it is necessary to have both lumbar and femoral bone tissue not compromised in quantity and quality.

Limitations are to be noticed. ANNs analysis is particularly suitable for clinical context characterised by vast samples with several variables of different clinical significances. The findings of this study are to be also validated in this type of context. This work deals with the capability of BSI to predict further fragility fracture. An ad hoc study to validate the ability of BSI to predict the first fragility fracture would complete the clinical validation trial.

Some conclusions arise from this study: firstly, lumbar and femoral BSI appear to be a useful index in characterising osteoporosis with fragility vertebral further fractures. It should be utilised for the identification of those patients prone to fragility fracture together with femoral and spine DXA indexes of low bone density. Secondly, ANNs Auto-CM is useful to understand the complexity of a chronic multifactorial disease like osteoporosis and to ameliorate the model of prediction of the severe consequences of osteoporosis, particularly further vertebral fragility fractures.

## Supporting information

S1 File Description of the methodology of TWIST algorithm system. (DOCX)

## Abbreviations

ANNs: artificial neural network analysis
BMI: body mass index
DXA: dual X-ray absorptiometry
BMC: bone mineral content
BMD: bone mineral density
BSI: bone strain index
SD: standard deviation
SDI: spine deformity index
VFs: vertebral fractures
LBMC/BMD: lumbar spine BMC/BMD
Neck_BMC/BMD: femoral neck BMC/BMD
FTot_BMC/BMD: total femoral BMC/BMD
LBSI: lumbar spine BSI
Neck_BSI: femoral neck BSI
FTot_BSI: total femoral BSI
