# Supplementary material for: Bone strain index as a predictor of further vertebral fracture in osteoporotic women: An artificial intelligence-based analysis
Source: PLoS One. 2021 Feb 8;16(2):e0245967. doi: 10.1371/journal.pone.0245967 (PMC7870050; doi:10.1371/journal.pone.0245967)
Supplement: S1 File — (DOCX) [file pone.0245967.s001.docx]

**Training With Input Selection and Testing (TWIST) Algorithm**

**TWIST system explanation**

The most relevant problem when using machine learning systems for pattern recognition is represented by the type of validation protocol and Inputs selection. Both problems may be expressed as follows:

1. How to create a valid couple of training-testing set which is statistically representative of the given problem;
2. How to choose the lowest number of input features that are able to maximize, in a blind test, the dependent variables accuracy.

The TWIST algorithm is a feasible way to approach both problems.

1. **Training and testing subsets optimal records distribution**

Each strategy for the distribution of the record dataset in a training, testing or a validation set is always performed with a random division of each record (observation) of the source dataset.

The aim for a random assignment is releted to statistics: as the source dataset corresponds to all the knowledge available for a specific assigned problem, we need to create two separate subsets of data which are more or less statistically. As a consequence, the training session will be a satisfactory set for the learning machine, and testing session results will be representative of the machine learning skill to generalize for the entire dataset. If from one side this principle is valid, still it is neither the only one and, possibly, nor the best. In fact, the aim of random criterion is that of optimizing the subsequent cost function:

(1) *f_1_*($d_{\Gamma}^{[tr]}$)*f_2_* ($d_{\Gamma}^{[ts]}$)*f_0_* ($d_{\Gamma}^{[global]}$);

in which *f_1_*($d_{\Gamma}^{[tr]}$) and *f_2_* ($d_{\Gamma}^{[ts]}$) = probability density function of testing - training subset, respectively; *f_0_* ($d_{\Gamma}^{[global]}$) = probability density function of the whole dataset.

This signify that the random criterion is aimed at generating two subsets with approximately the same probability density function; at the same time, both subsets should be statistically equivalent to the whole dataset.

The random criterion attempts to estimate the cost function expressed in the equation (1). Nevertheless, in order to enhance this cost function we should also consider every potential combination of each record within the two subsets; therefore, for any combination, we should measure and compare the probability density function of the single subsets.

Given a specific dataset *D* _Γ_ of N records, the number of samples *d_Γ_* composed of K possible records is obtained by:

$$\left(\begin{matrix} N \\ K \end{matrix}\right)$$

By changing K, you have:

(2) $\sum_{K=0}^{N} \left(\begin{matrix} N \\ K \end{matrix}\right)=2^{N}$ (*)

(*) the search space = 2^N^, despinte the satisfactory space is equal to $\frac{2^{N}-2}{2}$.

As a consequence, a couple of training and testing sets characterizes a possible solution$x=\left(D_{\Gamma}^{[tr]},D_{\Gamma}^{[ts]}\right)$, provided by the vector:

(3)

To further enhance the cost function showed in the equation number (1), we introduce an evolutionary algorithm whose population expresses different hypothesis after each generation about the division of the general dataset into 2 different subsets. Going more in detail, at any generation each subject of the genetic population suggests which record of the entire dataset have to be allocated into the subset A or into the subset B. Technically this is quite easy, as each individual of the genetic population is considered a vector of N Boolean values (1 or 0), in which N is the number of records included in the whole dataset.

Now, the main problem is to identify an appropriate fitness function that is capable of evaluating the validity of each hypothesis, according to the equation number 1.

To improve these constraints we used two autonomous Supervised Neural Networks (SNNs): usually, we use a Multilayer Perceptron (Back Propagation based). The evaluation of the fitness of each hypothesis has five steps independent among each other; every time each subject of the genetic population suggests its hypothesis of dividing the global dataset into two subsets (subset A and B):

1. The first SNN (SNN_A) is started and trained with the use of subset A, then it is stopped when the training error is minimized;
2. The trained SNN_A is applied blindly on the subset B, and its accuracy is registered;
3. The SNN_B, which is fully independent from the SNN_A), is then initialized and trained using the subset B, and again it is stopped once the training error is minimized;
4. The trained SNN_B is blindly applied to subset A, and its accuracy is registered;
5. The minimum value of the SNN_A and SNN_B accuracies is assigned as fitness of the hypothesis of splitting, and is created by any individual of the genetic population.

The different steps from 1 to 5 are called “Fitness Evaluation”, and are performed for any individual of the genetic population, at each generation of the evolutionary algorithm.

The following is the flow chart of the Training & Testing Optimization algorithm (T&T):

- Initialization of the Genetic Population
- Evolutionary Loop
  1. Fitness Evaluation of the possibility (hypothesis) of dividing each individual of the genetic population at the generation (n) (From step 1 to step 5);
  2. Production of crossover and offspring;
  3. Application of random mutation;
  4. New population setup;
  5. If the average fitness continues to grow up the algorithm start from the beginning, otherwise it terminates;
- Saving of the subset A and B presenting the best fitness.

**2) Choosing the minimum number of input features that are capable to maximize the dependent variable prediction accuracy**

Feature selection methods can be created using two different approaches that are based on whether variables selection is performed independently or dependently from the learning algorithm used to create the inductor. The “filter” approach is aimed at selecting the best attribute subset by evaluating its importance based on available data. The “wrapper” approach, on the other side, requires that the best attribute subset selection takes place considering as relevant those attributes allowing the induction algorithm to reach a better performance.

Our Input Selection (I.S.) algorithm operates as an evolutionary wrapper system that answer for the need of reducing data dimensions by extracting the lowest number of variables needed to control the “peaking” phenomenon and, simultaneously, preserving the highest information available. IS is an evolutionary system builtedn on the algorithm called GenD . It is capable to weigh the various variables of the dataset, instead of burdening the inducer to understand which of them are important. It works as a specific evolutionary wrapper system for the extraction of feature subsets. Differently from a filter system it uses a similar learning algorithm for the evaluation and selection of each feature [2].

The IS optimized sets are created considering only a specific subset of variables. For a specific dataset $D_{\Gamma}$ of *N* records and *M* variables, usuallt divided into a training set $D_{\Gamma}^{[tr]}$ and a testing set $D_{\Gamma}^{[ts]}$, we may have $\left(\begin{matrix} M \\ H \end{matrix}\right)$ possible samples $D'_{\Gamma}$, with corrispondent $D'_{\Gamma}^{[tr]}$and $D'_{\Gamma}^{[ts]}$, of *H* extracted variables; according to the variance of *H*, it is also$\sum_{H=0}^{M} \left(\begin{matrix} M \\ H \end{matrix}\right)=2^{M}$. An inducer $\Omega_{D_{\Gamma}^{'\text{ }[tr]},A,F,Z}\left(\cdot\right)$ can be created for each sample, by selecting an induction algorithm $A$, with its specific configuration parameters $F$and its parameters $Z$ for the initialization.

It is then possible to select variables to get the best performance, in a mode that is transposed respect T&T, for a specific couple of training and testing sets. By varying the input variables we may assume that the virtuousness of the results for each classifiers or predictors depends on whether the selected variables, putted together, are relevant or not relevant. It is not possible to know, a priori, the specific cause of the variable's relevance or not; they can be considered irrelevant by the system as they show redundant information, or because they may not contain information at all, or they present confusing unclear information or noise. Whatever the reason, we have no need to include such variables. As in the T&T algorithm, such operation of analyzing dataset variables before creating the inducer is another kind of data mining, that completes the extraction of the maximum amount of information that are contained whitin the variables.

Technically, IS is obtained with a GenD algorithm that consideres, as space of solutions, the $\sum_{H=0}^{M} \left(\begin{matrix} M \\ H \end{matrix}\right)=2^{M}$ possible combinations of variables, by using the subsequent two-symbol alphabet:

$$\Phi_{IS}=\left\{\text{ }\phi_{rel},\phi_{irrel}\right\}$$

In which the first symbol ($\phi_{rel}$) representing a variable that belongs to the set of relevant variables $V_{rel}$, and the second ($\phi_{irrel}$) representing a variable that belongs to the set of non-relevant variables $V_{irrel}$. A possible solution $x=\left(V_{rel},V_{irrel}\right)$ is therefore a couple of relevant plus irrelevant variable sets, being provided by the vector:

$$x=\left(V_{rel},V_{irrel}\right)=\left[x_{1},x_{2},\cdots,x_{M}\right]\in\begin{matrix} \begin{matrix} \Phi_{IS}^{M} & & & x_{i}\in\Phi_{IS} \end{matrix} & \end{matrix}$$

Similarly to the T&T, a *preliminary* and a *computational phase* are used to assess the parameters of the fitness function and to extract the most important training/testing set variables. A conventional back propagation ANN is used as the fitness function inducer $\Omega_{D_{\Gamma}^{[tr]},A,F,Z}\left(\cdot\right)$. This is arranged during the preliminary phase, and then used during the folklowing computational phase with fixed configuration and initialization parameters.

GenD that is applied to IS proposes other additional alternatives to that of selecting the best variable set, with a fitness that is less or equivalent to the best. These can be selected as a practical library of input selections that can be used when the best selection of variables is not available or is not convenient, for economic or different reasons.

As typically occurs for genetic wrapper systems, IS may exhibit a longer computational time, but also shows some advantages. In comparison to decision tree algorithms, IS shows greater performance robustness to the occurrence of several irrelevant features. However, compared to naive-Bayes algorithms, certain robustness to the existence of correlated features may be observed. IS receives the gradient based neural networks robustness, as for example back propagation, as well as the flexibility of evolutionary algorithms in order to sightsee the space fior the program solutions.

In order to integrate our IS algorithm with the T&T algorithm we modified the structure of the single genetic population individuals. This combination will generate a more complex algorithm that is able at searching for the best distribution of the whole sample into two subsets with the minimum of input features necessary for the best pattern recognition. We called this new algorithm as TWIST (Training With Input Selection and Testing). The integration is quite easy: in T&T algorithm each subject of the genetic population is a vector of N component of Boolean values, in which N represents the number of the variables of the whole dataset: in the case in which the value of a generic vectorcomponent is 1, the record is registered into the subset A; otherwise, when the value is 0, the record is therefore saved in the subset B.

When considering the I.S. algorithm, on the other side, each subject of the genetic population must be a vector of M component of Boolean values, in which M is the number of all the input variables: whenever the value of a generic vector component is 1, the corresponding input feature is registerd into both subset A and B, otherwise if the value is 0 the specific input feature is cancelled.

As a consequence, in TWIST algorithm each genetic population individual will be defined by two vectors, each one of a specific and different length:

1. A first vector that shows its hypothesis about which one of the records (*N*) must be stored within the subset A or subset B;
2. A second vector that shows its hypothesis about which inputs (*M*) must be used for the two subsets and which one must be cancelled.

After this change, the TWIST algorithm works similarly to the already described T&T algorithm.

Finally, after this evolution, the TWIST is able to generate two different subsets of data with a very similar probability distribution density and with the minimal number of variables needed for pattern recognition.
